# Association Between Vitamin D, Reproductive Hormones and Sperm Parameters in Infertile Male Subjects

**DOI:** 10.3389/fendo.2018.00607

**Published:** 2018-10-16

**Authors:** Rehana Rehman, Salima Lalani, Mukhtiar Baig, Iman Nizami, Zohaib Rana, Zohair Jamil Gazzaz

**Affiliations:** ^1^Department of Biological & Biomedical Sciences, Aga Khan University, Karachi, Pakistan; ^2^Department of Clinical Biochemistry, Faculty of Medicine, Rabigh, King Abdulaziz University, Jeddah, Saudi Arabia; ^3^Medical Student, Aga Khan University, Karachi, Pakistan; ^4^Department of Medicine, Faculty of Medicine, Rabigh, King Abdulaziz University, Jeddah, Saudi Arabia

**Keywords:** infertility, vitamin D, 25OHD, testosterone, FSH, LH

## Abstract

**Background:** The prevalence of infertility and vitamin D deficiency is common in Pakistan. Therefore, our study aims were to assess and compare Vitamin D; 25-hydroxyvitamin (25OHD) and reproductive hormone levels in male fertile and infertile subjects with normal and abnormal sperm parameters. Furthermore, the study is aimed to explore the association of 25OHD levels with these sperm parameters in a selected population of Karachi, Pakistan.

**Methods:** The cross-sectional study was carried out from August 2016 till December 2017, 313 study subjects were recruited from an Infertile Clinic from Islamabad, Pakistan, and the general population. First, we took the couples' history of parenting and then carried out a semen analysis and infertile and fertile male subjects were then subgrouped into “normal” and “altered sperm parameter/s.” Forward linear regression was done for selection of 25OHD as a significant predictor of sperm parameters.

**Results:** The median values of the total count, motility, morphology as well as serum 25OHD were significantly higher in the group with “normal” (186) as compared to subjects (127) in “abnormal sperm parameters” group. The 25OHD levels were significantly high in males with “normal sperm parameters”; 80.90 ± 23.33 nmol/L vs. “altered sperm parameter/s,” 64.68 ± 24.21 nmol/L (mean ± SD) with *p* < 0.001. Serum testosterone level had a significant positive correlation with 25OHD while LH had a significant negative correlation with 25OHD (*p* < 0.001), and FSH level had a non-significant negative correlation with 25OHD. Results of regression model showed one unit increase of motility would give 0.15-unit positive significant impact on 25OHD; 20% variation in 25OHD was explained by the total count, motility, and morphology, while the model was adjusted for BMI.

**Conclusion:** The impact of 25OHD levels on sperm parameters can be emphasized on the basis of detection of its high serum levels in “normal” subjects in both fertile as well as infertile males in comparison to subjects that had altered sperm parameters; total sperm count, motility, and normal morphology. The considerably positive association between 25OHD, testosterone, total count, motility, and morphology further accentuates its impact on normal spermatogenesis and the male reproductive functions required for acquiring fertility.

## Introduction

The existence of Vitamin D metabolizing enzymes and its receptors in male and female reproductive tissues indicate a role for vitamin D in human reproduction (1). The presence of the Vitamin D receptor (VDR), CYP2R1, CYP27B1, and CYP24A1 on the head and mid-piece of human sperm specifies that there is some role for Vitamin D 25OHD in sperm functionality, but that function is not fully understood. The VDR and these enzymes are also expressed in elongated spermatids, epididymis, seminal vesicle, and prostate (2).

The importance of 25OHD in human reproductive physiology has been further studied by proving the presence of the VDR on human sperm and noticing a significant decrease in sperm motility, total number of motile sperm, amount of sex hormone binding globulin, and testosterone/estradiol ration in 25OHD deficient males as compared to 25OHD sufficient males (3–5).

A recent study in Iran reported that almost half of the study population had 25OHD insufficiency and deficiency. Additionally, they reported no significant association between 25OHD and semen parameters and reproductive hormones in normospermic men while it had a positive relationship with sperm motility in the OAT cohort (6). Tirabassi et al. described a positive and significant effect of 25OHD concentration on the sperm motility (7). Another study reported that the deficient levels of serum 25OHD were linked to worse sperm parameters (8). Several studies have reported the positive correlation between 25OHD and free testosterone (FT) and total testosterone (TT) levels among healthy men (9, 10), while a study reported no significant correlation between 25OHD and sex hormones (11).

Evidently, the significance of the relationship between serum 25OHD levels and male fertility is inconclusive, but there is a clear association. In a population such as ours in which 25OHD deficiency is common, and about 20% suffer from infertility (12, 13), there is a need to explore whether this deficiency or insufficiency has an impact on normal reproductive function.

Our study aims were to compare 25OHD and reproductive hormone levels in male fertile and infertile subjects with normal and abnormal sperm parameters (sperm count/ejaculate, motility, and morphology) and observe the association of 25OHD levels with these sperm parameters to assess the possible role of 25OHD levels on normal reproductive functions thus leading to fertility.

## Subjects and methods

### Study design and population

The present cross-sectional study was carried out from August 2016 till December 2017 and 380 subjects were recruited. Before starting this research, we had prior information on the prevalence of male infertility to be 25–30%, by taking this probability we had drawn a sample size of 323 patients at 95% confidence interval with 5% margin of error by the given formula.

$$\begin{array}{l} n=\frac{Z_{\alpha /2}^{2}Pq}{e^{2}}=\frac{{\left(1.96\right)}^{2}\left(0.3\right)\left(0.7\right)}{{\left(0.05\right)}^{2}}\\ \;=323\;where\;\alpha =Confidence\;interval,p\\ \;=Probibality\;ofincidence\;and\;e=errorM\text{​}\arg\text{​}in \end{array}$$

**Figure 1:**
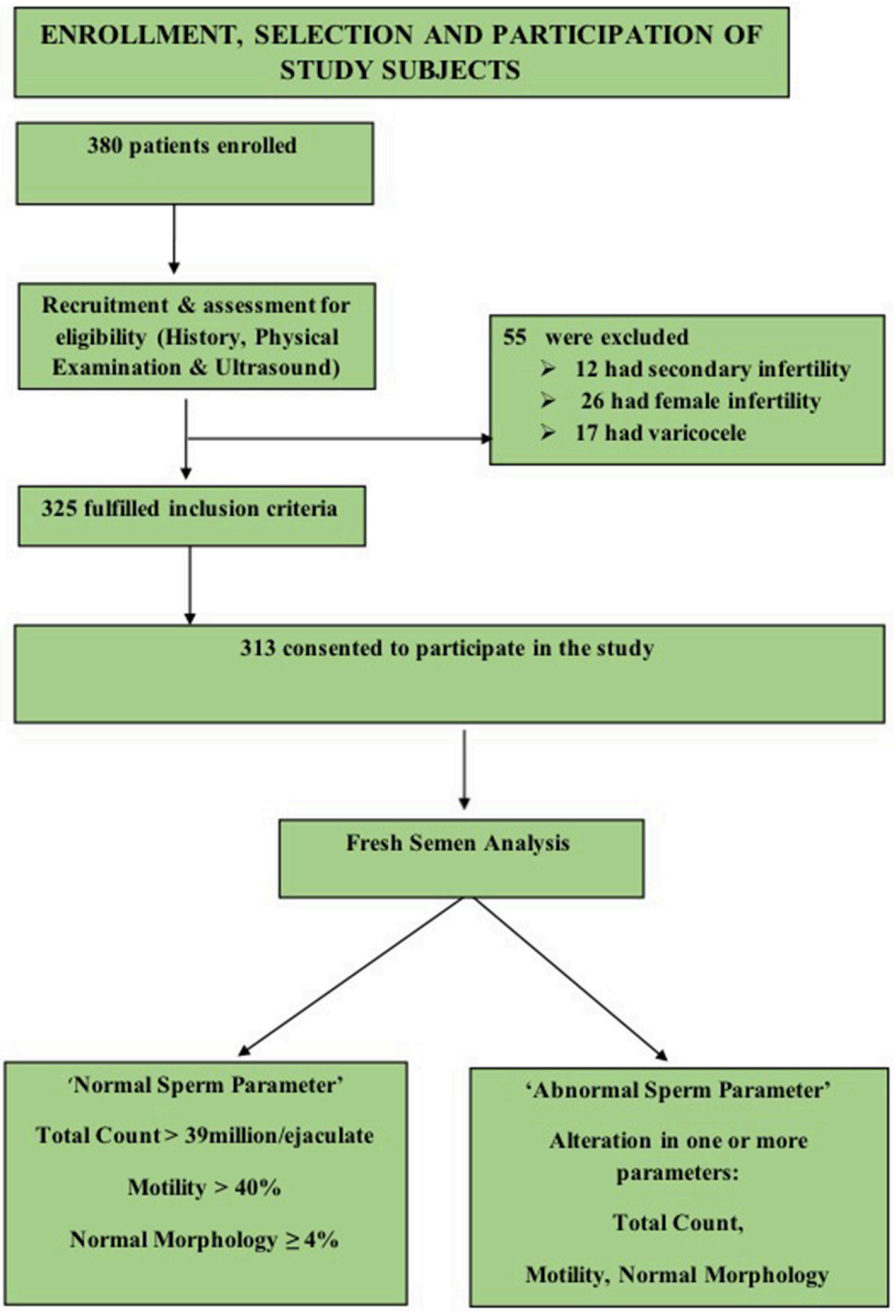
Flow chart showing enrollment, selection and participation of subjects.

A convenient sampling of 380 males was done, 67 subjects were excluded on the basis of criteria enlisted in Figure 1, and a final sample of 313 was achieved.

### Study protocol

The approval for this study was given by the Institutional Ethical Review Board. All subjects were asked to complete written and informed consent.

### Inclusion criteria

All male subjects with age ranging from 25 to 55 years during the study period who consented to take part and were not suffering any chronic disease or any endocrinal problems were included. The fertile participants were recruited from the general population. The group comprised of healthy males; without any history of fertility problems and whose spouses conceived within 1 year of unprotected intercourse (14). In addition to the fertile males, all the males referred to infertility clinic, were from couples who had failed to conceive after 1 year of regular unprotected intercourse, (14) with primary infertility and exception of female cause of infertility were included (a detailed history about the type and cause of infertility helped in exclusion of secondary infertility in males and female causes of infertility).

The study population thus comprised of 135 fertile and 178 infertile male subjects. The general physical examination, estimation of height, weight, and calculation of Body Mass Index (BMI) was followed by examination of testis, epididymis, vas deferens, and varicocele by (Valsalva maneuver). The genitourinary ultrasound was then performed.

### Exclusion criteria

Subjects who had the female cause of infertility, suffered from secondary infertility, had; cryptorchidism, testicular trauma, orchitis, and testicular hypotrophy were excluded. Moreover, subjects who were on 25OHD therapy, receiving testosterone or thyroxin replacement therapy and calcium supplementation were also eliminated. Subjects with cancer, epilepsy, diabetes, parathyroid gland disease, hypertension, malabsorption, gastric bypass, celiac disease, and inflammatory bowel disease and with a poor general health status were also excluded.

In order to avoid variation of semen analysis results obtained from different laboratories, assigned laboratory technicians performed fresh semen analysis of all patients. They were requested to provide semen samples by masturbation after three to five days of abstinence. All samples were saved in sterile containers, and World Health Organization guidelines were followed to examine the samples.

The fertility status of study participants in our study was based on semen parameters according to (WHO criteria “2010”) i.e., “had total sperm number (TC) >39 million per ejaculate, total sperm motility (Progressive and Non-progressive) measured within 60 min of collection of more than 40%, and %normal morphology (MORPH) of ≥4% (15). The “normal sperm parameters” group included male subjects who had TC >39 million per ejaculate, MOT >40 % and MORPH of ≥4%. “altered sperm parameter group” comprised subjects who had variation in at least one of the above criteria.

In order to know the fertility status (sperm parameters) in both fertile and infertile male subjects, we categorized both fertile and infertile subjects further into normal (normozoospermia) and altered sperm parameter/s. Therefore, “normal” in both fertile and infertile subjects had total sperm count (TC) >39 million per ejaculate, total sperm motility (progressive and non-progressive) more than 40% (measured within 60 min after collection of sample), and normal morphology of ≥4%. The subjects (both fertile and infertile) whose semen analysis showed any abnormality in sperm parameters as defined by WHO criteria (15), (i) TC <39 million sperms per ejaculate (ii) <40% motility measured within 60 min of collection, and (iii) <4% (or 5th centile) comprised subgroup of abnormal sperm parameter/s.

### Procedures

Around ten milliliters of venous blood from the ante-cubital vein was collected in the morning from study subjects. Serum obtained by centrifugation of blood was stored at −70°C until further analysis. Serum FSH was determined by using a commercially available kit for Human FSH Enzyme Immunoassay (Kit Cat. No KAPD1288 by DIA source Immuno Assays S.A. Belgium). Serum LH estimation was made by using a commercially available kit for Human LH Enzyme Immunoassay (Kit Cat. No KAPD1289 by DIA source Immuno Assays S.A. Belgium). Serum total testosterone was determined by using a commercially available kit for Human Total Testosterone (TT) Enzyme Immunoassay (Kit Cat. No 11-TESHU-E01 “*in vitro* Diagnostic” the United States of America). For Elecsys FSH immunoassay, the inter assay CV, and intra assay CV were <6 and <3%, respectively and for Elecsys LH immunoassay the inter assay CV and intra assay CV were <6 and <5%, respectively. For TT immunoassay, the inter assay CV and intra assay CV were <6%.

The intra-assay and inter-assay coefficient of variation (CV) for 25OHD was 2.7 and 4.3%, respectively. The lowest limit of detection was 2.8 ng/ml. We adopted the “Institute of Medicine cutoff points for vitamin D levels classifying serum levels into sufficient (50–125 nmol/L), insufficient (25–50 nmol/L), deficient (<25 nmol/L)” (16).

### Statistical analysis

Data were stored and analyzed using IBM-SPSS version 23.0. Medians and interquartile ranges were reported for sperm parameters; mean comparison between two study groups was performed using the Mann-Whiney U test. Pearson correlation of vitamin D, 25OHD.was also done to see the direction of age, TC, MOT, MORPH, and Rapid Linear Progression (RLP). Forward Likelihood Ratio (LR) regression was done for the selection of significant predictors of 25OHD. In the groups categorized on the basis of cut off values of 25OHD mean values compared using ANOVA, *post hoc* Tukey HSD test was then applied. All *p*-values < 0.05 were taken as significant.

## Results

**Figure 2:**
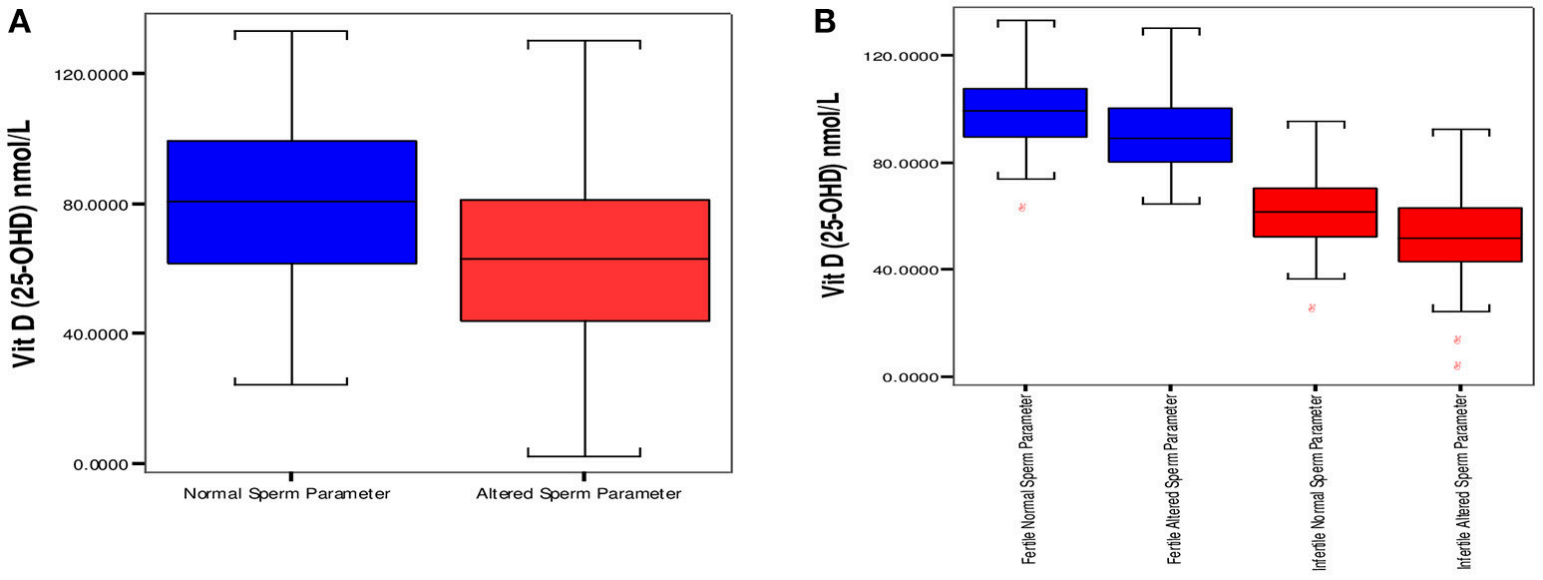
Distribution of vitamin D between groups having “normal” and “altered sperm parameter/s.” **(A)** Box plot of median 25 and 75th percentiles showed dissimilar distribution of vitamin D between normal and altered sperm parameters. **(B)** Comparison of Vitamin D among fertile and infertile groups having “normal” and “altered sperm parameter/s.” Normal sperm parameters = total sperm count > 39 milion sperm per ejaculate, motility > 40%, and normal morphology ≥ 4%. Aitered sperm parameter = total sperm count < 39 million sperm per ejaculate, motility < 40% and normal morphology < 4% (at least one parameter is altered).

On the basis of stratification done on sperm parameters in the study, there were 186 normozoospermic males (normal sperm parameter/s), and 127 subjects had one or more than sperm parameters altered (“altered sperm parameters”). Normozoospermic (93) male subjects were fertile and 93 reported to be infertile. There was a significantly high proportion of “altered sperm parameter/s” in infertile (85) as compared to fertile subjects (42). The 25OHD levels were significantly high in males with normal sperm parameters; 80.90 ± 23.33 nmol/L vs. altered sperm parameter/s, 64.68 ± 24.21 nmol/L (mean ± SD) with *p* < 0.001. Comparison of 25OHD levels in both groups is shown in Figure 2A.

The comparisons of median and interquartile range of male subjects with “normal” and “altered sperm parameters/s” in Tables 1A,B shows that the median values of serum 25OHD were significantly lower in the group which had altered TC/(MOT) and/or MORPH. In accordance with that serum FSH, LH and testosterone levels were significantly lower in subjects with “altered sperm parameter/s” as compared to normozoospermic (“normal sperm parameter”) group of subjects.

**Table 1A:** Comparison of study variables in groups characterized by normal and abnormal sperm parameters.

	**Normal sperm parameters (186)**		**Abnormal sperm parameter/s (127)**		
	**Median**	**IQR**	**Median**	**IQR**	***P*-value**
Age (Yrs.)	36	8	34	9	0.025
Total sperm count, (million per ejaculate)	110	60	38	55	0.000
Motility (%)	71	5	45	55	0.000
RLP (%)	1	3	0	1	0.000
MORPH (%)	6	6	2	1	0.000
Vitamin D (25-OH) (nmol/L)	80.8	37.525	63.175	37.625	0.000
FSH mIU/ml	4	3.7635	3.5	3.6689	0.007
LH (mIU/ml)	7.2235	3.8	6.9	3.2	0.266
Testosterone (ng/ml)	5.807	4.877	4.743	4.491	0.071

*Groups formed on the basis of semen analysis performed during the study*.

*Normal Sperm Parameters: total sperm count > 39 million sperm per ejaculate*.

*motility > 40%, and normal morphology ≥ 4%*.

*Altered Sperm Parameter Group had at least one of the following*:

*total sperm count < 39 million sperm per ejaculate*.

*motility < 40%, and normal morphology < 4%*.

*p < 0.05 considered significant using Mann Whitney U test*.

*RPL, Rapid Linear Progression; MORPH, Morphology; FSH, follicle stimulating hormone; LH, luteinizing hormone*.

**Table 1B:** Comparison of fertile and infertile subgroups having “normal” and “altered sperm parameters.”

**Variables**	**Fertile (135)**					**Infertile (178)**				
	**Normal sperm parameter (*****n*** = **93)**		**Altered sperm parameter (*****n*** = **42)**		***P*-value**	**Normal sperm parameter (*****n*** = **93)**		**Altered sperm parameter/s (*****n*** = **85)**		***P*-value**
	**Median**	**IQR**	**Median**	**IQR**		**Median**	**IQR**	**Median**	**IQR**	
Age (Years)	37	9	37	10	0.084	33	6	33	6	0.654
Sperm count (Million spermatozoa/ejaculate	110	60	50	52	0.000	110	60	20	48	0.000
Motility (%)	71	5	50	30	0.000	71	5	35	47	0.000
RLP (%)	1	3	0.5	1	0.083	1	3	0	1	0.000
MORPH (%)	6	6	2	2	0.000	6	6	1	2	0.000
Vitamin D (25-OH) (nmol/L)	99.22	18.15	89.35	19.92	0.001	61.72	18.15	51.85	20.1	0.000
FSH mIU/ml	6.35	4.6	6.85	2.20	0.418	3.106	2.2	2.8	1.27	0.024
LH (mIU/ml)	9.7	2.13	9.5	2	0.457	6	4.4	6.39	2.84	0.030
Testosterone (ng/ml)	8.04	1.40	8.18	0.87	0.867	3.32	3.20	3.78	2.363	0.146

*Normal sperm parameters: total sperm count > 39 million sperm per ejaculate*.

*motility > 40%, and normal morphology ≥ 4%*.

*Altered sperm parameter group had at least one of the following*:

*total sperm count < 39 million sperm per ejaculate*.

*motility < 40%, and normal morphology < 4%*.

*RPL, Rapid Linear Progression; MORPH, Morphology; FSH, follicle stimulating hormone; LH, luteinizing hormone*.

*p < 0.05 considered significant using Mann Whitney U test*.

On the basis of 25OHD levels subjects 1(0.5%), 17(9.1%), and 168 (90.3%) were declared deficient, insufficient and sufficient in “normal sperm parameter group” (16). In the “altered sperm parameter/s” group, estimation of 25OHD levels showed; 4 (3.1%), 36 (28.3%), and 87 (68.5%) subjects labeled as deficient, insufficient and sufficient.

The comparison of 25OHD (nmol/L, mean ±SD) in normal and “altered sperm parameter/s” of fertile subjects was 99.64 ± 13.86 vs. 90.60 ± 14.04 (*p* < 0.001). In infertile subjects significantly high values of 25OHD (nmol/L, mean ±SD) was observed in “normal” infertile; 62.15 ±13.86, in comparison to 51.87 ± 16.77 in infertile “altered sperm parameter/s.” The comparison is shown in Figure 2B.

**Figure 3:**
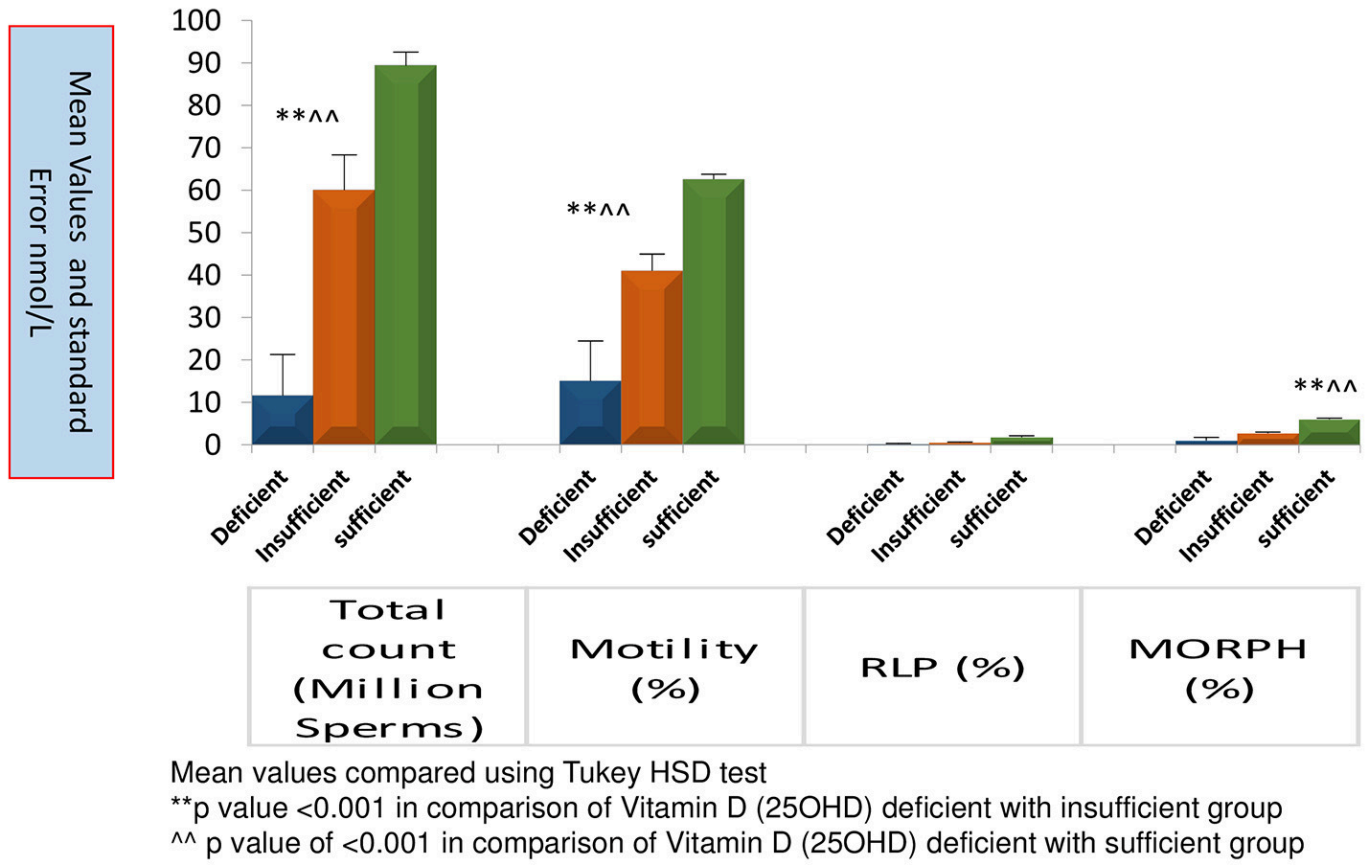
Comparison of sperm parameters according to Vitamin D status. Mean value compared using Tukey HSD test.

Figure 3 represents significantly high TC, MOT and MORPH in 25OHD sufficient group as compared to the other groups. All sperm parameters were significantly compromised when males had deficiency or insufficiency of 25OHD.

Table 2 reports the correlation value of 25OHD with all Sperm Parameters and Male Reproductive Hormones. A positive association of 35.1, 25, 42.9 and 30.8% was obtained for age, TC, MOT, and MORPH, respectively. A strong positive correlation of 0.637 was observed between testosterone (ng/ml) and 25OHD with a significance level of < 0.01 while LH had a significant negative correlation with 25OHD (*p* < 0.001), and FSH level had a non-significant negative correlation with 25OHD.

**Table 2:** Correlation of vitamin D (25OHD) with sperm parameters and male reproductive hormones in the study cohort.

**Parameters**		**Vitamin D**	***p*-value**
Age (Years)	r-value	0.351	<0.05^*^
Total sperm Count (Million spermatozoa /ejaculate	r-value	0.250	<0.05^*^
Motility (%)	r-value	0.429	<0.05^*^
Morphology (%)	r-value	0.308	<0.05^*^
RLP (%)	r-value	0.067	0.234
FSH (mIU/ml)	r-value	−0.097	0.085
LH (mIU/ml)	r-value	−0.321	<0.001^*^
Testosterone (ng/ml)	r-value	0.637	<0.001^*^

* *P < 0.05 was considered significant using Pearson's correlation*.

*RPL,Rapid Linear Progression; MORPH, Morphology; FSH, follicle stimulating hormone; LH, luteinizing hormone*.

Table 3 depicts the results of the regression model obtained for the factors (TC, MOT, and MORPH) that are influenced by 25OHD using the forward linear regression method. As observed, one unit increase of 25OHD led to 1.37, 1.00, and 0.001 increases in the TC, MOT, and MORPH, respectively, whereas RLP seemed unaffected and was excluded due to the *p*-value of more than 0.05. Adjusted R-square value suggests that 25OHD is responsible for 6, 18, and 0.1% of the variance of the total count, motility, and morphology.

**Table 3:** Regression analysis showing relationship of Vitamin D (25OHD) with sperm parameters in the study cohort.

	**Regression coefficients**			
**Parameters**	**Beta coefficient**	**Standard error**	***t*-value**	***p*-value**
Total count	−0.004	0.012	−0.374	0.708
Motility	0.151	0.030	5.083	<0.001^*^
Morphology	20.148	15.705	1.283	0.201

*Dependent variable: Vitamin D, model was adjusted with BMI*.

*Adjusted R-square = 20%*,

* *p < 0.05 considered significant*.

## Discussion

The present study found a significant decrease in 25OHD levels in subjects that had altered sperm parameters; TC, MOT, and MORPH. These results are similar to several other studies (5, 17). Conversely, few studies have reported contradictory results (4, 8, 18), and one of the reasons could be that in most of the studies their all study participants were healthy control from the general population as opposed to our selected group of patients. However, we are unable to find the exact pathophysiological mechanism of action of 25OHD that was responsible for abnormal sperm parameters, thus causing infertility. Nevertheless, VDR is found in male reproductive tissues such as human testis, ejaculatory tract, and mature spermatozoa (2), and this indicates a role of 25OHD in human reproduction. Recently, Fu et al. stipulate that 25OHD deficiency reduces testicular weight and sperm quality; moreover, there is suppression of testicular germ cell production and a decline in mature seminiferous tubules percentage in mice. They also reported that 25OHD deficiency reduces the enzymes for testosterone synthesis and, consequently, serum, and testicular testosterone levels were decreased (18).

The present study found a significantly positive association between 25OHD and total sperm count and motility. Similar results were obtained by other studies (3, 7). These results are further supported by the findings of the VDR and 25OHD metabolizing enzymes in the sperm head and the male reproductive tract. Low sperm motility has also been reported with deficient or insufficient 25OHD levels (17, 18), which is also evident in our findings. Hammoud et al. observed that low 25OHD levels were associated with decreased total sperm count and progressive motile sperm count but did not affect sperm concentration, progressive motility and normal morphology (4). Literature indicates that 25OHD raises intracellular calcium levels and motility of sperm; it also brings the acrosome reaction in mature spermatozoa, and there was a positive association between serum 25OHD levels and sperm motility (3). On the basis of which it can be suggested that deficiency, as well as insufficiency, disturb the sperm motility.

It is reported in the literature that quality and count of sperms are dependent on many parameters such as optimal level of enzymes, hormones, and antioxidants (19). Vitamin D 25OHD level may not affect sperm production directly but can be a major contributing factor when fertility is concerned.

In the present study, we observed a high sperm count in subjects with sufficient levels of 25OHD. Zhu et al. reported serum 25OHD level was positively associated with progressive sperm motility and TC in infertile patients (11). He did not find any significant relationship between serum 25(OH)D3 or 1,25(OH)2D3 with reproductive hormones, despite low 25OHD levels among different categories of infertile subjects and its relationship with TC and progressive motility. It seems the relationship of 25OHD with sperm parameters is independent of the reproductive hormones.

In infertile males with severe oligoasthenoteratozoospermia along with deficient vitamin D 25OHD levels, pregnancy outcomes have been found to be compromised if serum concentration should fall below 50 nmol/L (20). In our study, we observed that increased TC, motility and normal morphology was significantly high in the group of male subjects who had sufficient 25OHD levels. Numerous studies (human and animal) have reported the importance of 25OHD in male fertility. It has been postulated that 25OHD actions are mediated by the presence of VDR and metabolizing enzymes in the adult male reproductive tract, male germ cells, and Leydig cells, which are the major places of testosterone production in men (2, 21).

Blomberg Jensen et al. reported that serum calcium is imperative for spermatogenesis, sperm motility, and the acrosome reaction and 25OHD is a key regulator of the calcium and phosphate homeostasis (3). It may be hypothesized that 25OHD has a dual role in human reproduction and exert its function by maintaining calcium and phosphate homeostasis and by influencing sex hormone synthesis (3, 10).

We observed higher levels of testosterone, FSH and LH levels in the fertile cohort especially with “normal sperm parameters.” The role of 25OHD on reproductive hormones can be supported by a significant positive correlation of testosterone with 25OHD. Our results are consistent with a few other studies that found a positive correlation between 25OHD and testosterone (10, 22, 23) while inconsistent with a few others (7, 24). A Korean study reported that low 25OHD level (<20 ng/ml) was linked with an augmented risk of testosterone deficiency (10). Based on the positive relationship between 25OHD and testosterone, it can be assumed that harmful effects of higher or lower 25OHD level on the process of fertility could be interceded by the testosterone. Researchers have reported higher FSH and LH levels in infertile patients (11) while few studies described no correlation between 25OHD and reproductive hormones (3, 6). The high LH and FSH levels in “normal sperm parameters” and the correlation between 25OHD and reproductive hormones signify that 25OHD l plays a crucial role in the fertility mechanism.

We have found a significant association of 25OHD with sperm motility. It is not clear how the 25OHD influences the sperm motility and morphology. It is done either by direct action or under the influence of reproductive hormones. Still, there is a gap in the knowledge regarding its exact mechanism of action on sperm parameters. Several aspects are uncovered and thus not understandable. Sun et al. proposed that the relationship between 25OHD and sperm motility and morphology might not be due to the direct action of 25OHD, but it exerts this action via ion homeostasis (25).

As far as clinical studies are concerned, available data is limited. An experimental Chinese study reported that in idiopathic oligoasthenozoospermia, 25OHD treatment significantly increased the proportion and count of progressively motile sperm and conception rate (26). A double-blinded randomized clinical trial reported the absence of any significant treatment effect of vitamin D3 on serum testosterone levels in a group of healthy men (27). Another clinical trial found significant improvement in metabolic profile, serum testosterone levels, and erectile function in middle-aged vitamin D, 25OHD-deficient men after 1-year treatment with vitamin D (28). Additionally, a recent triple-blinded, randomized clinical trial reported that semen quality parameters did not change in 25OHD-insufficient infertile men despite the intake of high doses of vitamin D preparations. However, there was an increase in the chances of live birth and serum inhibin B in oligozoospermic males as well as an increase in spontaneous pregnancies as well (29). Such results show the clinical importance of vitamin D supplements in vitamin D-deficient infertile male subjects. However, the discrepancy in clinical data demands further longitudinal experimental studies on a larger scale with a large sample size to explore the precise role of 25OHD in male infertility.

## Limitations

The study was conducted on a small cohort as far as subgrouping of infertile subjects in which lifestyle influences, the status of smoking, screening by DNA Fragmentation Index (DFI), calcium and phosphorus levels, and Parathyroid hormone status were not estimated. Furthermore, our study neither noted the seasonal differences in 25OHD levels among either group nor the influence of 25OHD on seasonal semen variations. Moreover, we did not look at the genetic polymorphism of Vitamin D receptors or Vitamin D binding protein responsible for the decreased 25OHD levels.

## Strengths

In the era of increased deficiency of 25-OHD, our study high lights its importance in acquiring normal sperm parameters thus leading to fertility. The study also compares semen analysis performed in both fertile and infertile males.

In a study done to observe effects of vitamin D levels on pregnancy outcome after intracytoplasmic sperm injection (ICSI), researchers observed that “deficiency of 25-OHD in females hinders the accomplishment of optimal endometrial thickness required for implantation of embryo after ICSI.” The improvement in vitamin D status can thus improve success results in assisted reproductive clinics (30). However, the literature on Vitamin D leaves current knowledge “uncertain” partly because studies on males or females often fail to consider the total fertility picture where variables across the gender profile need to be factored in if we are to obtain the clearest picture.

## Conclusion

The impact of 25OHD levels on sperm parameters can be emphasized on the basis of detection of its high serum levels in “normal” subjects in both fertile as well as infertile males. Our results showed significantly lower levels of 25OHD in infertile men who had “altered sperm parameter/s” stated in terms of reduced TC, motility, and/or normal morphology. The considerably positive association between 25OHD, testosterone, total sperm count, motility, and morphology likewise accentuates its impact on normal spermatogenesis and the male reproductive functions required for acquiring fertility. In light of our results, it can thus be concluded that reduced 25OHD levels in infertile male subjects disturb normal physiological mechanisms required for being fertile, and it is possible that corrected or improved vitamin D (25OHD) level in males could result in successful conception. Therefore, its inclusion in the treatment regime of infertility should be considered. It will now be of interest to evaluate the effect of Vitamin D supplementation in this population of Pakistani men and stratify semen profile outcomes according to the degree of deficiency.

## Ethics statement

The approval for this study was given by the Institutional Ethical Review Board of Infertile Clinic, Islamabad, Pakistan. An informed written consent was obtained from all participants of the study.

## Author contributions

RR conceived the research idea, got the data collected, and drafted the manuscript. SL and IN analyzed data and contributed to manuscript writing. ZG and MB performed the literature search, designed the paper outline and final revision and drafting of the manuscript. ZR contributed to the database search, data analysis and result compilation. All authors participated in the research significantly and critically appraised, revised and approved the manuscript.

### Conflict of interest statement

The authors declare that the research was conducted in the absence of any commercial or financial relationships that could be construed as a potential conflict of interest.
